# Diagnostic assessment by dynamic contrast-enhanced and diffusion-weighted magnetic resonance in differentiation of breast lesions under different imaging protocols

**DOI:** 10.1186/1471-2407-14-366

**Published:** 2014-05-24

**Authors:** Hongmin Cai, Lizhi Liu, Yanxia Peng, Yaopan Wu, Li Li

**Affiliations:** 1 School of Computer Science & Engineering, South China University of Technology, Guangzhou 510006, People’s Republic of China; 2 Sun Yat-sen University Cancer Center, State Key Laboratory of Oncology in South China, Imaging Diagnosis and Interventional Center, Guangzhou 510060, People’s Republic of China; 3 Department of Radiology, The Third Affiliated Hospital of Sun Yat-sen University, Guangzhou 510630, People’s Republic of China

**Keywords:** Diffusion-weighted imaging, Breast mass, Quantitative morphology and texture features, Computer-aided diagnosis, Classifier, Feature subset selection

## Abstract

**Background:**

The apparent diffusion coefficient (ADC) is a highly diagnostic factor in discriminating malignant and benign breast masses in diffusion-weighted magnetic resonance imaging (DW-MRI). The combination of ADC and other pictorial characteristics has improved lesion type identification accuracy. The objective of this study was to reassess the findings on an independent patient group by changing the magnetic field from 1.5-Tesla to 3.0-Tesla.

**Methods:**

This retrospective study consisted of a training group of 234 female patients, including 85 benign and 149 malignant lesions, imaged using 1.5-Tesla MRI, and a test group of 95 female patients, including 19 benign and 85 malignant lesions, imaged using 3.0-Tesla MRI. The lesion of interest was segmented from the raw image and four sets of measurements describing the morphology, kinetics, DW-MRI, and texture of the pictorial properties of each lesion were obtained. Each lesion was characterized by 28 features in total. Three classical machine-learning algorithms were used to build prediction models on the training group, which evaluated the prognostic performance of the multi-sided features in three scenarios. To reduce information redundancy, five highly diagnostic factors were selected to obtain a compact yet informative characterization of the lesion status.

**Results:**

Three classification models were built on the training of 1.5-Tesla patients and were tested on the independent 3.0-Tesla test group. The following results were found. i) Characterization of breast masses in a multi-sided way dramatically increased prediction performance. The usage of all features gave a higher performance in both sensitivity and specificity than any individual feature groups or their combinations. ii) ADC was a highly effective factor in improving the sensitivity in discriminating malignant from benign masses. iii) Five features, namely ADC, Sum Average, Entropy, Elongation, and Sum Variance, were selected to achieve the highest performance in diagnosis of the 3.0-Tesla patient group.

**Conclusions:**

The combination of ADC and other multi-sided characteristics can increase the capability of discriminating malignant and benign breast lesions, even under different imaging protocols. The selected compact feature subsets achieved a high diagnostic performance and thus are promising in clinical applications for discriminating lesion type and for personalized treatment planning.

## Background

There is a growing clinical interest in developing noninvasive tissue characterization methods that can be used early in the course of diagnosis to assess risk and to guide subsequent treatment by allowing clinicians to conduct a therapy on an individual [1,2]. Magnetic resonance imaging (MRI) methods such as dynamic contrast-enhanced (DCE) and diffusion-weighted (DW) methods are among those of interest, as they provide noninvasive digital biomarker measurements of tissue properties that are highly relevant to the assessment of tumor progression and/or responses [3]. DW-MRI generates images that are sensitive to water displacement at the diffusion scale and quantifies such diffusion according to a quantitative index reflecting the apparent freedom of diffusion (apparent diffusion coefficient (ADC)). DW-MRI has been reported to achieve higher detection rates than mammography [4,5], and can easily be adopted as an adjunction for standard clinical imaging protocols [1,6]. Preclinical and clinical reports show that ADC reflects regional cellularity, which results in significantly lower values in malignant tumors than in benign breast lesions or normal tissue due to an increasing restriction on the extracellular matrix and a higher fraction of signal from intracellular water [7-9]. It has been reported recently that the mean ADC value of malignant tumors is reduced compared with that of benign lesions and normal tissue in vivo DW-MRI, and thus this technique is promising for the characterization of breast lesions [10]. However, false negatives and underestimation of cancer spread were also observed owing to artifacts based on bleeding and tumor structure [11].

DCE-MRI, on the other hand, uses the serial acquisition of images during and after the injection of an intravenous contrast agent. It has been shown to reflect tumor vascularity and to achieve higher sensitivity than other imaging modalities in delineating invasive lobular carcinoma, which is not evident on conventional imaging [12,13]. DCE-MRI has high sensitivity to breast cancer detection (89–100%), while DW-MRI shows good performance in monitoring response after therapy [14].

A recognized weakness of both DCE-MRI and DW-MRI is their low specificity in discriminating between benign and malignant lesions (37–86%) [15-17]; therefore, biopsy tests are frequently adopted as a remedy, which inevitably introduce sampling errors. Recent studies focus on comparing and retrospectively integrating the contributions from different modalities by combining the merits of different modalities [18,19]. This work has highlighted the potential of combining multi-modality characteristics to differentiate the core of the tumor from peritumoral tissues and normal tissues, and thus to provide richer information on lesion status than individual imaging modalities [20,21].

During the image interpretation phase, well-trained and experienced radiologists are needed to read an MRI image. However, even well-trained experts may have high inter-observer variation rates, so computer-aided diagnosis (CAD) is necessary to help radiologists in detecting and classifying breast cancer [22]. Recently, several CAD approaches have been studied to minimize the effects of operator-dependent errors that are inherent in magnetic imaging, and to increase diagnostic sensitivity and specificity [23]. For example, feasibility and efficiency of CAD systems for breast cancer detection and classification by the use of ultrasound images has been demonstrated by others [22,24]. A CAD system using selected features from a set including lesion shape, texture, and enhancement kinetics was built and tested using a back-propagation neural network [25]. As much as 65–90% of the biopsies turned out to be benign; therefore, a crucial goal of breast cancer CAD systems is to distinguish benign from malignant lesions to reduce false positives. Many machine learning techniques such as linear discriminant analysis, support vector machines (SVM) and artificial neural networks have been studied for mass detection and classification [26].

We, together with other researchers, have shown that combining different modalities, such as DCE-MRI and DW-MRI, can dramatically increase the power in discriminating pathologically verified breast masses [21,27-29]. For example, Nie et al. reported six features selected from morphology and texture descriptors by an artificial neural network and developed a classification model for computer-aided diagnosis [30]. Partridge et al. investigated the discrimination power of ADC from DW-MRI and demonstrated an improved positive predictive value of breast lesions, which was calculated for DCE-MRI alone [14].

However, these earlier studies mainly concentrated on patients collected under similar protocols. Therefore, the obtained prognostic models, as well as the selected prognostic factors, were not validated extensively. We conducted an independent validation study concerning breast mass discrimination on two patient datasets collected under different imaging conditions. We focus on evaluating the potential discriminatory power by integrating DCE-MRI with DW-MRI. Twenty-eight distinct features were estimated to comprehensively characterize the segmented mass. Three scenarios were analyzed to resolve three major concerns. 1) Does the high diagnostic power reported still hold in an independent validation study? 2) Does a full characterization of breast mass improve diagnostic performance? 3) Can a compact feature set achieve good diagnostic performance? Our studies have given positive answers to these three questions through extensive experiments using standard classification models including SVM [31-33], *k*-nearest neighbors (KNN) [34] and Random Forest [35]. Finally, five highly prognostic factors that are invariant under various imaging conditions were found. These factors are valuable in clinical practice since they can provide accurate information solely dependent on tumor characteristics.

## Methods

### Clinical cases

This retrospective study was approved by the institutional review board (IRB) and ethics committee of Sun Yat-sen University Cancer Center, China. Neither patient approval nor informed consent was required for review of medical records or images. Informed consent was signed and obtained from all patients before biopsy or surgery prior to procedures as a daily practice. This study consisted of two groups of patients with lesions detected on breast MR images. These data were collected at the Sun Yat-sen University Cancer Center. Between September 2008 and December 2011, a total of 234 consecutive female patients were enrolled in the first group (training group), including 85 benign and 149 malignant lesions. All of the patients in the training group underwent a breast MRI examination in a 1.5-Tesla system. The mean age of these women was 46 years (ranging from 18 to 78 years). Between January 2011 and December 2011, a total of 93 consecutive female patients with 18 benign and 75 malignant lesions were enrolled in the second group (test group). The patients in the test group underwent a breast MRI examination in a 3.0-Tesla system. The mean age of these 93 women was 45 years (ranging from 16 to 74 years).

The breast MRIs were interpreted using assessment and breast density categories established by the American College of Radiology and reported in the Breast Imaging Reporting and Data System (BI-RADS) by two radiologists who had 3–10 years’ experience in breast imaging. The entire breast images, breast tissue or lesions were classified as per the following assessments: need additional imaging evaluation (category 0); negative (category 1); benign finding (category 2); probably benign finding with a recommendation for additional imaging or biopsy (category 3); suspicious (category 4); or highly suggestive of malignancy (category 5). All of these cases were selected by experienced radiologists based on the following inclusion criteria. 1) Multiple breast MRI imaging sequences, including T1- and T2-weighted images, pre- and post-contrast images, DCE-MRI and DW-MRI, can be loaded simultaneously. 2) Nodal or mass lesions on breast MRI classified as category 2–5. 3) All malignant (category 4–5) and probably benign lesions (category 3) on MR images were verified by open surgical biopsy or fine needle biopsy, and all benign lesions (category 2) on MR images were verified by biopsy or follow-up at least 2 years after MRI examination.

Patients were excluded from the trial for any of the following criteria: 1) history of previous breast biopsy within a week or any therapy on breast lesions before MRI examination; 2) lesions not visible in any sequences on breast MRI imaging; 3) lesions classified as category 3–5 could not be verified by histopathology. Characteristics and histopathology of the lesions in the two groups are summarized in Table 1.

**Table 1:** Data summary

	**Training group**		**Testing group**	
Benign lesions^#^	1.3(0.5-3.0)cm		1.8(0.5-9.0)	
Malignant lesions^#^	2.8(1.5-5.0)cm		2.6(0.5-5.5)cm	
	Number	Percentage	Number	Percentage
**BI-RADS assessments**				
category 2	30	12.8	17	18.3
category 3	41	17.6	41	44.1
category 4	98	41.8	27	29.0
category 5	65	27.8	8	8.6
**Malignant lesions**	**149**	**63.68**	**75**	**80.6**
Invasive ductal carcinoma	120	51.3	62	66.7
Intraductal carcinoma	17	7.26	9	9.7
Ductal carcinoma in situ	4	1.7	1	1.1
Mucinous carcinoma	3	1.28	2	2.1
Medullary carcinoma	1	0.43	0	0
Others	4	1.71	1	1.1
**Benign lesions**	**85**	**36.32**	**18**	**19.4**
Fibroadenoma	26	11.11	6	6.4
Fibrocystic changes	24	10.26	3	3.2
Fibroadenosis	3	1.28	3	3.2
Intraductal papilloma	4	1.71	3	3.2
Hyperplasia	3	1.28	1	1.1
Phyllodestumor	2	0.85’	1	1.1
Adenomyosisepithelioma	1	0.43	0	0
Inflammation	1	0.43	1	1.1
Follow-up	21	8.97	0	0

Note: ^#^summarizes the median size of the lesions, whose range is listed by parentheses.

Characteristics and histopathology of benign and malignant breast lesions.

### Image acquisition

The patients in the training group underwent MRI in a 1.5-Tesla superconductive magnetic system (GE, Signa, HDx). The patients in the test group underwent MRI in a 3.0-T superconductive magnetic system (Siemens, Trip Tim). A breast-specific 4-channel phased-array surface coil was used. The images consisted of axial cross-sectional and sagittal T2-weighted fast spin-echo, sagittal T1-weighted non-fat-suppressed, T1-weighted fat-suppressed DCE before and after contrast material administration, and DW sequences prior to gadolinium-based contrast material injection in axial orientation. DCE MR imaging data were acquired using an MRI-specific automatic power injector (Medrad, Pittsburgh PA) to inject 0.1 mmol/kg body weight contrast medium gadolinium diethylenetriaminepenta-acetic acid (Gd-DTPA) with a hand venipuncture technique at a rate of 3 ml/s. Saline, 10 ml at 3 ml/s, was then injected to wash the tube.

For 1.5 Tesla MR imaging, DW-MRI was performed using single-shot echo planar imaging, fat suppression, b values of 0 and 800 s/mm^2^, 5000/75 (repetition time msec/echo time msec), 5-mm section thickness, a 30 × 30-cm field of view, a 256 × 256 matrix, 0 mm section gap, and 130 sec acquisition time. DCE MRI was obtained using 3D Fast FSPGR pulse sequence, with repetition time msec/echo time msec of 5.5/2.6, a matrix of 288 × 288, and nine postcontrast acquisitions. Temporal resolution was 59 seconds per dynamic acquisition.

For 3.0 Tesla MR imaging, DW-MRI was acquired using a spin-echo echo-planar imaging, fat suppression, b values of 0 and 800 s/mm^2^, 5400/86 (repetition time msec/echo time msec), 5-mm section thickness, a 30 × 30-cm field of view, a 192 × 192 matrix, 1 mm section gap, and 130 sec acquisition time. DCE MRI was obtained using a (fast low angle shot three dimensional imaging) FL3D sequence, with repetition time msec/echo time msec of 4.15/1.55, a matrix of 256 × 205, and nine postcontrast acquisitions. Temporal resolution was 270 seconds per dynamic acquisition.

### Lesion image segmentation

The manual segmentation was first performed by an experienced radiologist and optimized by a two-step approach through which we incorporated fuzzy c-means clustering [36] and a gradient vector flow snake algorithm [37], the details of which we have reported elsewhere. This segmentation was performed piece by piece and the lesion region of interest in each piece was visually assessed by the radiologists.

### Pictorial characterization of the segmented lesion from MR images

Once a segmented lesion image was obtained, one can characterize its pictorial properties by using a standard technique for image analysis. In our study, four groups of features were designed to reflect the distinct characteristics of the mass images, including kinetics, morphology, texture and DW-MRI features.

The morphological group of features is traditionally used in clinical practice and it mainly summaries the one-dimensional statistics. Eleven morphological features were estimated for each segmented lesion. The features of the group include compactness, spiculation, extent, elongation, solidity, circularity, entropy of radial length distribution, fractal, heterogeneity, area, and eccentricity. Texture features are widely used in the pattern recognition domain to assist in differentiating imaged objects automatically, such as natural scenes versus non-natural scenes. They have also been widely used to analyze breast cancer images to discriminate abnormalities from normal masses [38]. Fundamentally, texture features are high order statistics of the image. Thirteen texture features were estimated on the segmented lesion through its gray level co-occurrence matrix [39].

The texture features included angular second moment, contrast, correlation, inverse difference moment, average of sum, variance of sum, entropy of sum, entropy, average of difference, variance of difference, entropy of difference, measurement of correlation 1 information, and measurement of correlation 2 information [40]. Readers are referred to Additional file 1 for detailed definition of the features.

Both the early-phase enhancement (EPE) and the signal enhancement ratio (SER) [41] were estimated to represent the kinetic behavior of the lesion signal intensity before and after the injection of Gd-DTPA. The time-intensity profile for the classification of breast cancer on dynamic magnetic resonance images through an artificial neural network was used by the radiologist to achieve a better diagnostic accuracy [42]. The kinetic features included EPE and SER, defined by [43]

$$\begin{array}{l} \mathrm{EPE}=\frac{I_{0}-I_{\mathrm{init}}}{I_{\mathrm{init}}}\%\\ \;\mathrm{SER}=\frac{I_{0}-I_{\mathrm{init}}}{I_{\mathrm{last}}-I_{\mathrm{init}}}\% \end{array}$$

where *I*_0_, *I*_*init*_ and *I*_*last*_ represent the signal intensity in pre-contrast, first post-contrast and last images, respectively.

The discrimination capability of ADC has been validated, and its expression is shown to be significantly lower in malignant tumors than in benign breast lesions or normal tissue in DW-MRI [6-8,11,44,45]. It has been shown to be an effective parameter in distinguishing malignant from benign breast lesions [8]. Here, we used the ADC value to characterize the lesion segmented from the DW-MRI [28,46]. The DW-MRI intensity of each lesion was first dichotomized into a low and high value by comparing the breast tissue with the corresponding background. The averaged ADC values were computed to represent the characteristics of DW-MRI.

**Figure 1:**
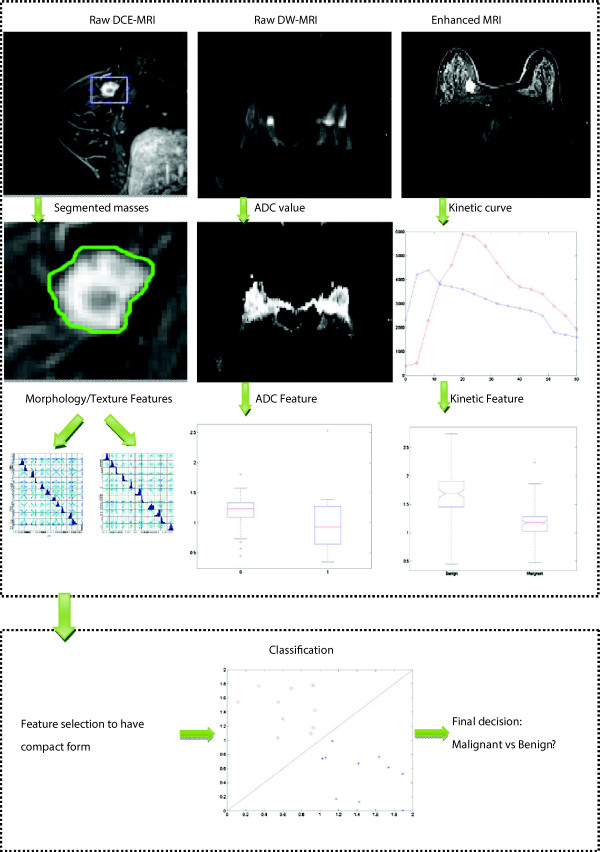
**Overview of the analysis pipeline.** Raw DCE-MRI is segmented to have suspicious breast mass, on which morphological and texture features are estimated. The ADC map is calculated on DWI-MRI to have the ADC feature. Kinetic curve is obtained on the enhanced image of DCE-MRI and then kinetic features are estimated. Features are extracted and selected within the combined features, and used by the classifier to predict whether the sample is malignant or benign.

The four groups generated 28 *features* for each lesion. All the features obtained were extracted by two radiologists who had 10 years’ experience in interpreting breast MRIs. They were blind to the histological results. The status of breast masses enrolled in the study were all verified histopathologically, or confirmed in at least the following two years. The systematic pipeline, consisting of four steps including image segmentation, feature calculation, feature extraction and classification, is summarized in Figure 1.

### Classification performance of individual features

We first assessed the overall classification performance of each individual feature in classifying lesion types. For each individual feature, the best cut-off value with which to differentiate benign from malignant lesions was first estimated on the training group through analyzing the receiver operating characteristics (ROC). The best cutoff value was defined as the value corresponding to the highest average of sensitivity and specificity. This value was then evaluated on the test group to validate its diagnostic performance. To remove the bias due to different magnetic field levels as well as inter-observer interpretations, the two groups were normalized using a standard z-transformation. The area under the maximum likelihood-estimated binormal ROC curve (AUC) was used as an index of performance. Features whose AUC was larger than 0.5 were further analyzed using an independent-samples *t*-test to compare malignant with benign. A *p*-value of less than .05 was considered to indicate a significant difference. Software (Matlab, version R2011b; MathWorks Com. Ltd., Boston, MA, USA) was used for all data analysis.

### Classification performance of multi-sided features

It has been shown by ours research as well as in earlier studies that an individual feature is less effective in the characterization of breast lesions than multiple features combined [21,27-29,46]. The evaluation of multiple features combined together in discriminating benign lesions from malignant ones is usually considered a binary classification problem. The status of the lesions is the observed outcome, on which a supervised classification model can be built. Consequently, the models obtained are then applied to evaluate the ability of each feature class (morphology, texture, kinetic texture and kinetic signal intensity) and to classify each lesion as benign or malignant. The features corresponding to each feature class are used as inputs to the classifier individually and in combination. To achieve extensive comparisons, three classical classification models including SVM [31-33], KNN [34] and Random Forest [35] were used in our study. We tested the classification performance of the features individually as well as in combination by using the three classification models. Therefore, the bias caused by the classification scheme could be largely ameliorated and the diagnostic potential of the features could be ascertained through extensive experiments. A short introduction to the three classification models is provided in Additional file 2.

Though each segmented lesion was fully characterized by multi-sided descriptions, a redundant feature set will inevitably result, and deteriorate classification performance. To alleviate this drawback, a recently reported method for feature selection, called the Local Hyperplane-based RELIEF (LHR) feature weighting scheme, can be used to select a subset of features with high prognostic values [47-49]. The feature selection scheme of LHR is chosen owing to its good performance, in particular its immunity to classification models. We then tested the well-selected features using the three classification models to evaluate their discrimination power. A short introduction to the LHR model is provided in Additional file 3.

## Results

### Diagnostic performance of each feature individually

Among the 28 estimated features, eleven of them achieved large AUC (>0.5), as shown in Table 2. The top three features are ADC, SER and sum average. The values of the corresponding AUC are as high as 0.85, 0.71 and 0.70, respectively. However, a common drawback of these three factors is their low sensitivity measurement, making them infeasible in clinic practice.

**Table 2:** Diagnostic performances of the features

**Feature name**	Parameter distribution^*^		***P-*** **value**^**#**^	**Specificity**	**Sensitivity**	**Accuracy**	**AUC**
	**Benign**	**Malignant**					
***Elongation***	***0.84 ± 0.13***	***0.87 ± 0.11***	***0.39***	***0.11***	***0.89***	***74.19***	***0.58***
***ADC***	***1.04 ± 0.21***	***1.50 ± 0.43***	***0.00***	***0.67***	***0.92***	***87.10***	***0.85***
**SER**	1.20 ± 0.22	1.00 ± 0.50	0.01	0.33	0.97	84.95	0.71
**Correlation**	0.65 ± 0.16	0.60 ± 0.17	0.23	0.06	0.96	78.49	0.60
**Inertia**	1995.34 ± 1177.11	2773.68 1891.29	0.03	0.17	0.93	78.49	0.64
***Entropy***	***8.48 ± 1.30***	***7.90 ± 1.34***	***0.09***	***0.11***	***0.99***	***81.72***	***0.64***
**Inverse Difference**	0.10 ± 0.05	0.09 ± 0.03	0.22	0.11	0.97	80.65	0.65
***Sum average***	***310.21 ± 37.83***	***285.58 ± 34.20***	***0.01***	***0.11***	***0.99***	***81.72***	***0.70***
***Sum variance***	***9235.63 ± 2999.89***	***10078.03 ± 3168.82***	***0.29***	***0.00***	***1.00***	***80.65***	***0.57***
**Sum entropy**	6.76 ± 0.87	6.29 ± 0.90	0.04	0.11	0.99	81.72	0.66
**Difference average**	32.44 ± 10.15	38.73 ± 14.30	0.03	0.28	0.93	80.65	0.66
**Difference variance**	820.01 ± 486.86	1042.80 636.38	0.10	0.06	0.99	80.65	0.62
**Difference entropy**	5.40 ± 0.44	5.16 ± 0.47	0.04	0.11	0.99	81.72	0.66
**Information Correlation 1**	−0.58 ± 0.12	−0.61 ± 0.14	0.43	0.11	0.95	78.49	0.58

Note: 1. ^#^Computed with paired-sample *t*-test.

2.^*^The distribution of the variables are denoted in form of Mean ± Standard Deviation.

Statistical analysis of the independent 3.0-Tesla patients group. For each individual variable, its diagnostic performance is tested through ROC analysis on 1.5-Tesla patients group. The five variables (highlighted in italic) when combined together to consist of a highly diagnostic feature subset is shown to outperform over any individual variables in Table 3.

### Diagnostic performance of multi-sided features in combination

We considered three scenarios when evaluating the classification performance of multi-sided features in combination on the dataset. In the first scenario (scenario 1), we tested whether entire features achieved superior performance to individuals or combinations during diagnostic classification. In the second scenario (scenario 2), we tested whether ADC still possesses a high prognostic value when the magnetic field changed from 1.5-Tesla to 3.0-Tesla. In the third scenario (scenario 3), we tested whether carefully selected features achieved superior or comparable diagnostic performance to the entire feature set. Three conclusions were drawn with respect to the three scenarios.

### Scenario 1: Entire features outperform individual or combinations of features during diagnostic classification

The estimated feature groups described distinct characteristics of the breast lesions that thus had different discrimination powers. First, we investigated the discrimination power of each feature group individually and then compared them with their combinations. Since the morphological information was widely used in clinical practice, it was used as the borderline to compare with other feature groups. Different combinations of feature groups with morphological features were tested using the three classifiers and their average performance was also computed. The results are summarized in Table 3. When using morphological features alone, the classification of an independent dataset of patients showed a high specificity of 0.817 but a very low sensitivity of 0.278 (tested by SVM), which implied a low degree of true positive. Therefore it underestimated the possibility of malignant masses when using morphological information, resulting in a delay of clinical treatment. However, the combination of the morphological feature with texture features, kinetic features or both dramatically increased sensitivity. For example, the average sensitivity was increased from 0.445 to 0.518, 0.556, and 0.611 after combining morphological features with texture features, kinetic features and both, respectively. The corresponding AUCs were improved from 0.566 to 0.61, 0.681 and 0.689. Therefore, the characterization of breast masses in a multi-sided way would dramatically increase the sensitivity value by increasing true positives. Moreover, using the entire estimated feature set would dramatically increase the performance of the three classifiers, thus achieving the best results. For example, the maximum specificity and sensitivity values were 0.722 and 0.924, which were increased by 30% and 4.8% more than by using morphological, texture and kinetic features together, when using SVM on entire feature groups. On average, entire feature groups showed a higher performance in both sensitivity of 0.685 and specificity of 0.912 than any individual groups or their combinations. The two values were increased by 12.1% and 2.2% more than by using morphological, texture and kinetic features together.

**Table 3:** Diagnostic performances of the classification models

**Classifier**	**Feature subset**	**Specificity**	**Sensitivity**	**Accuracy**	**AUC**
**SVM**	Morphology	0.278	0.817	67.74	0.526
	Morphology + Texture	0.444	0.851	69.89	0.602
	ADC + SER	0.722	0.926	81.72	0.781
	Morphology + Kinetic	0.5	0.875	77.42	0.67
	*Morphology + ADC*	*0.611*	*0.903*	*81.72*	*0.739*
	Morphology + Texture + Kinetic	0.556	0.882	75.27	0.678
	Entire^*%^	*0.722*^*30%*^	*0.924*^*4.8%*^	*79.57*^*5.7%*^	*0.768*^*13.3%*^
**KNN**	Morphology	0.5	0.85	64.52	0.569
	Morphology + Texture	0.444	0.844	66.67	0.619
	ADC + SER	0.722	0.917	73.12	0.784
	Morphology + Kinetic	0.556	0.867	66.67	0.66
	*Morphology + ADC*	*0.611*	*0.892*	*74.19*	*0.794*
	Morphology + Texture + Kinetic	0.611	0.887	70.97	0.666
	Entire^*%^	*0.611*^*0%*^	*0.899*^*1.4%*^	*78.49*^*10.6%*^	*0.744*^*11.7%*^
**Random Forest**	Morphology	0.556	0.871	68.82	0.604
	Morphology + Texture	0.667	0.864	53.76	0.609
	ADC + SER	0.667	0.9	70.97	0.764
	Morphology + Kinetic	0.611	0.885	69.89	0.713
	*Morphology + ADC*	*0.667*	*0.91*	*78.49*	*0.8*
	Morphology + Texture + Kinetic	0.667	0.906	75.27	0.722
	Entire^*%^	*0.722*^*8.3%*^	*0.912*^*1%*^	*69.89*^*-7.2%*^	*0.787*_*9%*_
**Average**	Morphology	0.445	0.846	67.03	0.566
	Morphology + Texture	0.518	0.853	63.44	0.61
	ADC + SER	0.703	0.914	75.27	0.776
	Morphology + Kinetic	0.556	0.876	71.33	0.681
	*Morphology + ADC*	*0.630*	*0.873*	*78.13*	*0.778*
	Morphology + Texture + Kinetic	0.611	0.892	73.84	0.689
	Entire^*%^	*0.685*^*12.1%*^	*0.912*^*2.2%*^	*75.98*^*2.9%*^	*0.766*^*11.2%*^

Remark 1: Entire ^*%^refers to using entire feature set, i.e., Morphology + Texture + Kinetic + ADC, and the subscript ^*%^denotes the increased ratio from Morphology + Texture + Kinetic to Morphology + Texture + Kinetic + ADC.

Diagnostic performances of three classical classification models and their average on different feature subsets. Incorporation of the feature of ADC will dramatically increase the discrimination power of the classification models as well as their average.

### Scenario 2: ADC is highly diagnostic and can increase sensitivity when combined with other features

It has been reported that ADC is a very informative diagnostic variable [7-9]. The ADC is significantly lower in malignant tumors than in benign breast lesions or normal tissue in DW-MRI owing to its high cell density, caused by an increased restriction of the extracellular matrix and an increased fraction of signals from intracellular water. Similar observations were produced in our study. When using morphology and ADC features together, the classification performances of the three classifiers conducted on the independent group of patients beat all other possible combinations of morphology and other features, show in Table 3. The former achieved the highest AUC of 0.739, 0.794, and 0.8 after SVM, KNN and Random Forest, respectively. The average AUC of morphology plus ADC was 0.778, which was higher than that of morphology combined with texture (0.61), kinetic (0.681) or both (0.689). Further analysis shows that the good performance of ADC is due to its dramatic improvement in sensitivity, implying outstanding discrimination in malignant patients. When using features other than ADC, the sensitivity value ranged from 0.278 to 0.667. After incorporating ADC during classification, the range was greatly extended from 0.611 to 0.722. A simple *t*-test shows that the two groups are statistically different (*p*-value < 0.001), as shown in Table 3. Finally, adding ADC to all other features achieved superior performance to using the features without ADC. For example, when using morphology, kinetic and texture features together, the overall accuracies are 75.27% after SVM, 70.97% after KNN, and 75.27% after Random Forest. In comparison, the accuracy increased to 79.57% after SVM, 78.49% after KNN, and 69.89% after Random Forest. The suboptimal performance of Random Forest is mainly due to the classification scheme of the tree-like structure, which is sensitive to data variations between training and test data like ours. On average, the incorporation of ADC can dramatically increase the discrimination power compared with not using ADC in terms of sensitivity, specificity, accuracy and AUC from 0.611 to 0.685 (increase of 12%), 0.892 to 0.912 (increase of 2%), 73.84% to 75.98% (increase of 3%) and 0.689 to 0.766 (increase of 11%), respectively.

### Scenario 3: Carefully selected features achieved the best diagnostic performance

**Figure 2:**
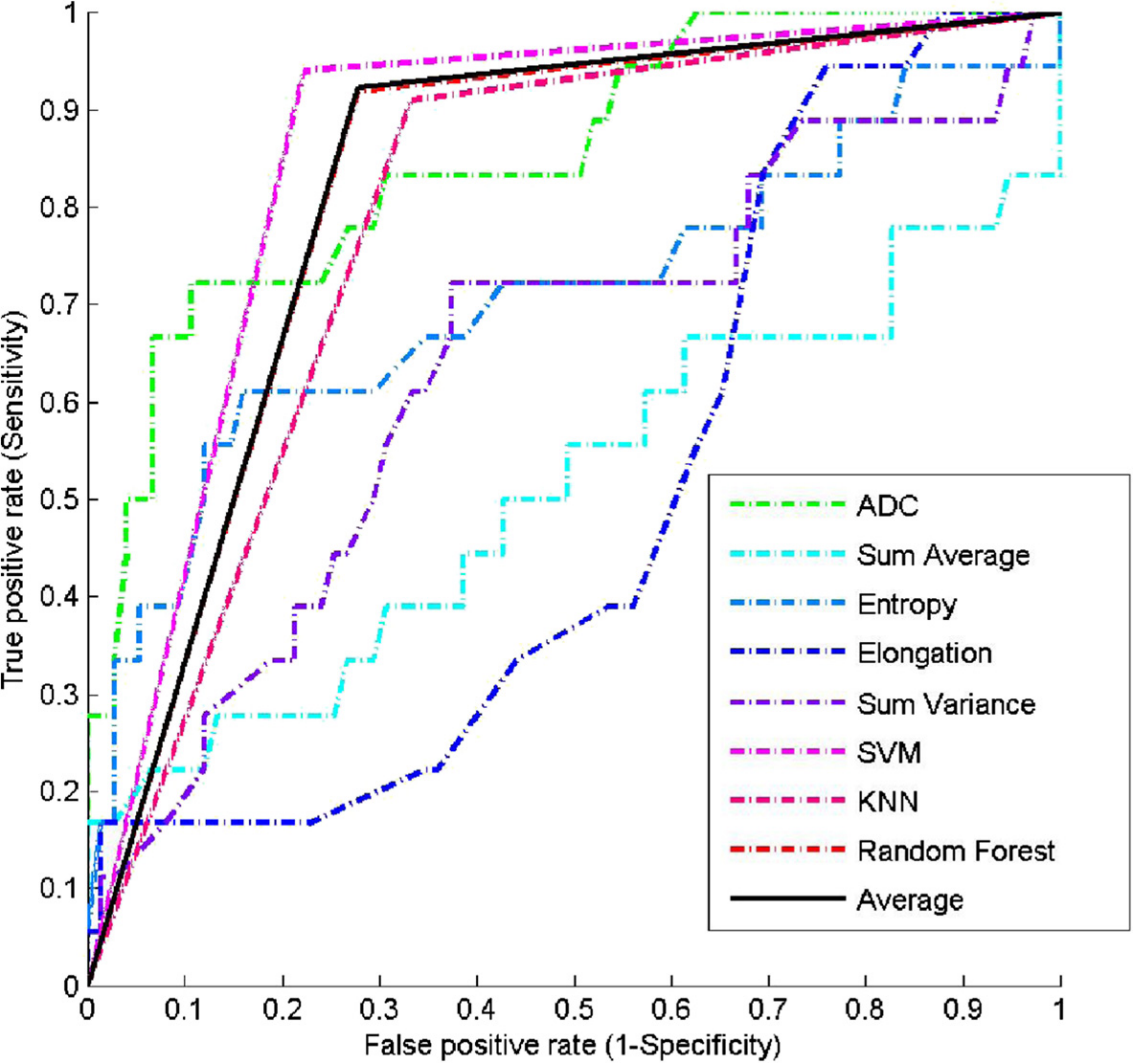
**Validations via ROC plot.** ROC plot of the carefully selected features from 1.5-Tesla patients in diagnostic prediction on 3.0-Tesla patients. For the individual features, thresholds were estimated from 1.5-Tesla patients and then were used on the independent 3.0-Tesla patients. The resulted ROC curves were plotted in dashed lines. The ROC curves for the selected prognostic features after SVM [31-33], KNN [34] and Random Forest, [35] were plotted in solid lines.

The estimated features were redundant in characterizing the lesion masses and therefore reduced the prediction performance of the three classifiers. A feature selection method reported recently, called LHR, uses a highly diagnostic yet compact feature subset [49]. The five features discovered include ADC, Sum Average, Entropy, Elongation and Sum Variance. The results of the classification performance on the selected feature subset are reported in Table 4. Both the AUC and accuracy of the selected features are better than for all features after using the three classification models. For example, the accuracies of SVM on the selected feature subset and on the all-feature set were 82.8% and 79.57%, respectively. The averaged AUC and accuracy on the selected features were 78.5% and 0.805, which is increased from 75.98% and 0.766 on all features. For clarity, the ROC curves for the three models after selected features in individually or their combinations are shown in Figure 2.

**Table 4:** Diagnostic evaluation of the selected features

**Selected feature**	**Criteria**	**Specificity**	**Sensitivity**	**Accuracy**	**AUC**
ADC	SVM [31-33]	0.778	0.94	82.8	0.809
Sum average	KNN [34]	0.667	0.91	78.50	0.815
Entropy	Random Forest [35]	0.722	0.92	74.19	0.791
Elongation					
Sum variance	**Average**	**0.722**	**0.923**	**78.50**	**0.805**

Evaluation of the discrimination power of five selected informative features through three classical classification models.

## Discussion

Both 1.5 T and 3.0 T systems are widely used in clinical practice. Magnetic power can influence the imaging parameters, such as signal-to-noise ratio, contrast-to-noise ratio, spatial resolution, and sequence acquisition time. Whether these changes in imaging protocol can influence the diagnostic performance of the classification models is rarely reported. The current study aimed to fill the gap by building a prognostic model on the training group of 1.5-Tesla patients and test it on the test group of 3.0-Tesla patients. Our multi-parametric model provided a high accuracy both in the 1.5-Tesla and 3.0-Tesla group. The results after the three well-designed scenarios demonstrate that diagnostic performance can be dramatically improved by incorporating multi-sided characterizations of breast lesions in MRI. The ADC parameter in particular shows a close relationship with lesion malignancy due to a high cell density, caused by an increased fraction of signals from intracellular water. This parameter, when combined with morphology and enhancement kinetic features, can increase both the specificity and sensitivity in discriminating lesion types, and thus is a promising candidate to provide supplementary assessment of lesion status.

Our study has some limitations. First, the databases of the 3.0-Tesla group were not large enough to allow the extraction of a strict statistical model. Considering that both 1.5-Tesla and 3.0-Tesla systems are widely used in clinical practice, it will be valuable to evaluate the diagnostic performance of MRI at 3.0-Tesla on larger sample sizes in future research. Second, the pictorial characteristics were estimated on 2D slices and currently we are working on 3D characterization of the lesions to obtain accurate volumetric measurements.

## Conclusions

The current study has highlighted the potential of combining DCE-MRI with DW-MRI to differentiate breast mass from normal via extensive validation. Our study demonstrates that diagnostic performance can be dramatically improved by characterization of breast lesions through the incorporation of multi-modalities of the MRIs, thus yielding better mass classification than with individually processed features of the two modalities. The ADC parameter is confirmed to have a high diagnostic value alone or in combination with other features and our analysis shows that its good performance is mainly due to improvements in specificity. Our study also reported a compact yet informative variable for diagnostic prediction that has the highest performance. This may be useful for building a CAD system combining of the ADC value, morphological, and DCE features to help radiologists in classifying breast lesions on MRI.

## Competing interests

The authors declare that they have no competing interests.

## Authors’ contributions

HM conducted the statistical analysis of the data and drafted the manuscript. LZ and YX collected the patient data, helped to analyze the data with relevant medical literature and revised the manuscript. LL supported the study coordination and YP designed the experiment. All authors read and approved the final manuscript.

## Pre-publication history

The pre-publication history for this paper can be accessed here:

http://www.biomedcentral.com/1471-2407/14/366/prepub

## Supplementary Material

Additional file 1 **Pictorial feature definitions.** Detailed definitions of the texture and morphological features used to characterize the lesion. Click here for file

Additional file 2 **Classification models. **A short introduction of the classification models used in our experiments. Click here for file

Additional file 3 **Feature selection model of LHR. **A simple explanation of the feature selection model of LHR. Click here for file
