# Supplementary material for: Diagnostic assessment by dynamic contrast-enhanced and diffusion-weighted magnetic resonance in differentiation of breast lesions under different imaging protocols
Source: BMC Cancer. 2014 May 24;14:366. doi: 10.1186/1471-2407-14-366 (PMC4036635; doi:10.1186/1471-2407-14-366)
Supplement: Additional file 1 — Pictorial feature definitions. Detailed definitions of the texture and morphological features used to characterize the lesion. [file 1471-2407-14-366-S1.docx]

**Appendix 1**

#### *Morphological measurements of breast lesions*

Once the segmented lesion image is obtained, one can estimate its morphological characteristics. Fundamentally, the morphological features estimate the first-order statistics of the image.

*p*_1_: Compactness

$p_{1}=\frac{P^{2}}{4\pi S}$,

where *P* and *S* are the perimeter length and area for a given breast MRI lesion contour, respectively.

*p*_2_: Spiculation

$p_{2}=\frac{1}{N}\sum_{i=1}^{N} \left|r_{i}-r_{i+1}\right|$,

where *N* is the number of pixels on the lesion contour and *r_i_* is the individual radial length.

*p*_3_: Extent

$$p_{3}=\frac{S}{S_{\text{box}}}$$

where *S*_box_ is the area of the smallest rectangle containing the given lesion contour.

*p*_4_: Elongation

$$p_{4}=\frac{\min(H,L)}{\max(H,L)}$$

where *H* and *L* are the vertical and horizontal lengths of the smallest rectangle containing the given lesion contour.

*p*_5_: Solidity

where *S*_convex_ is the area of the smallest convex polygon that can contain the given lesion contour.

*p*_6_: Circularity

$$p_{6}=\frac{1}{N-1}\sum_{i=1}^{N} r_{i}-\mu_{r}$$

where $\mu_{r}$is the average of *r_i_*.

*p*_7_: Entropy of radial length distribution

$$p_{7}=-\sum p(r_{i})\log(p(r_{i}))$$

where *p*(*r_i_*) is the probability density of a given *r_i_*.

*p*_8_:Heterogeneity

Fraction of pixels that deviate more than a certain range (10% defaults) from the average intensity.

*p*_9_:Fractal dimension

Fractal dimension=$\lim_{\varepsilon\to0}\frac{N_{\varepsilon}}{1/\varepsilon}$, where$N_{\varepsilon}$is the number of boxes of side length $\varepsilon$required to cover the lesion image.

*p_10_*:Area

Pixel numbers of the lesion region.

*p_11_*:Eccentricity

The ratio of the distance between the foci of the ellipse and its major axis length.

#### *Texture measurements of breast lesions*

To achieve comprehensive characterization of breast lesion morphology, 11 parameters were estimated automatically from gray-level co-occurrence matrix (GLCM) on the segmented lesion image. The GLCM estimates the second-order statistics of the image. Mathematically, the GLCM element over an image S is given as

$p(i,j)=\frac{\#\{[(x,y),(x+\Delta x,y+\Delta y)]\in S|f(x,y)=i,f(x+\Delta x,y+\Delta y)=j\}}{\#S}$，

where # represents the number of pixel-pairs. Let *N* be the number of distinct gray-levels in image S, and define,

$p_{x}(i)=\sum_{j=1}^{N} p(i,j)$, $i=1,2,\cdots,N$

$p_{y}(j)=\sum_{i=1}^{N} p(i,j)$, $j=1,2,\cdots,N$

$p_{x+y}(k)=\sum_{i=1}^{N} \sum_{j=1}^{N} p(i,j)$, $k=i+j=2,3,\cdots,2N$

$p_{x-y}(k)=\sum_{i=1}^{N} \sum_{j=1}^{N} p(i,j)$, $k=\left|i-j\right|=0,1,\cdots N-1$

The thirteen texture features are then calculated as

*f*_1_: Angular Second Moment

$f_{1}=\sum_{i} \sum_{j} p(i,j)^{2}$.

*f*_2_: Contrast

$f_{2}=\sum_{k=0}^{N-1} k^{2}p_{x-y}(k)$.

*f*_3_: Correlation

$f_{3}=\frac{1}{\sigma_{x}\sigma_{y}}\sum_{i=1}^{N} \sum_{j=1}^{N} (ij)p(i,j)-\mu_{x}\mu_{y}$,

where$\mu_{x}$and$\sigma_{x}$are the mean and standard deviations of *p_x_*, respectively; $\mu_{y}$and$\sigma_{y}$ are the mean and standard deviations of *p_y_*, respectively.

*f*_4_: Inverse Difference Moment

$f_{4}=\sum_{i} \sum_{j} \frac{1}{1+(i-j)^{2}}p(i,j)$.

*f*_5_: Sum Average

$f_{5}=\sum_{k=2}^{2N} kp_{x+y}(k)$.

*f*_6_: Sum Variance

$f_{6}=\sum_{k=2}^{2N} (k-f_{5})^{2}p_{x+y}(k)$.

*f*_7_: Sum Entropy

$f_{7}=-\sum_{k=2}^{2N} p_{x+y}(k)\log(p_{x+y}(k))$.

*f*_8_: Entropy

$f_{8}=-\sum_{i} \sum_{j} p(i,j)\log(p(i,j))$.

*f*_9_: Difference Average

$f_{9}=\sum_{i=1}^{N} (i-\mu)^{2}p_{x}(i)$,

where$\mu$is the average of *p*(*i*,*j*).

*f*_10_: Difference Variance

$f_{10}=\sum_{k=0}^{N-1} (k-d)p_{x-y}(k)$,

where *d* is the mean of *p_x-y_.*

*f*_11_: Difference Entropy

$f_{11}=-\sum_{k=0}^{N-1} p_{x-y}(k)\log(p_{x-y}(k))$.

*f*_12_: Information Measure of Correlation 1

$f_{12}=\frac{f_{9}+\sum_{i} \sum_{j} p(i,j)\log(p_{x}(i)p_{y}(j))}{\max(-\sum_{j} p_{x}(i)\log(p_{x}(i)),-\sum_{j} p_{y}(i)\log(p_{y}(i)))}$.

*f*_13_: Information Measure of Correlation 2

$f_{13}=\sqrt{1-\exp(-2(E-f_{8}))}$,

where $E=-\sum_{i} \sum_{j} p_{x}(i)p_{y}(j)\log(p_{x}(i)p_{y}(j))$.
