# Supplementary material for: Diagnostic assessment by dynamic contrast-enhanced and diffusion-weighted magnetic resonance in differentiation of breast lesions under different imaging protocols
Source: BMC Cancer. 2014 May 24;14:366. doi: 10.1186/1471-2407-14-366 (PMC4036635; doi:10.1186/1471-2407-14-366)
Supplement: Additional file 2 — Classification models. A short introduction of the classification models used in our experiments. [file 1471-2407-14-366-S2.docx]

***Appendix 2.***

#### *A short introduction of the classification models*

In this article we consider the lesion discrimination problem as a two-class pattern classification problem. We shall use a vector to denote the pictorial characterizations of components (in our experiments) for a segmented lesion. In our binary classification, each lesion was labeled by its pathological status, malignant or benign.

**Support Vector Machine** aims to minimize the bound on generalization error rather than traditional mean square error [53]. A SVM decision function $D(x)$ can be written as:

$$D\left( x \right)=\omega^{T}\varphi\left( x \right)+b$$

where parameter$\omega=\{\omega_{1},\omega_{2},\cdots\omega_{n}\}$ denotes the support vector. It is obtained through minimization of the following so-called structural risk function:

$$J\left( \omega,x \right)={\frac{1}{2}\omega}^{T}\omega+C\sum_{i=0}^{n} \xi_{i}$$

Subject to:

$$d_{i}D\left( x_{i} \right)\geq1-\xi_{i}$$

$$\xi_{i}\geq0,i=1,2,\cdots n$$

The value of is a user-specified positive parameter, and $\xi_{i}$ are slack variables.


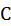


The model could be generalized to deal with nonlinear problem by transforming the input feature vector into a higher dimensional space through the underling nonlinear mapping $\varphi(x)$.


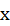


**K-nearest Neighbors (KNN)** algorithm is a famous instance-based learning algorithm [54]. It firstly computes an averaged Euclidean distance between a newly observed sample to its k nearest neighbors from each class, and then assign the class labelto which the distance is minimized


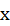

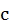

$$c=\arg\min_{c\in\{benign,maligant\}} \sum_{i=1}^{k} |\left| x-x_{k} \right||$$

where$x_{k}$is the nearest neighbors for the sample .


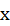


**Random Forests** are an [ensemble learning](http://en.wikipedia.org/wiki/Ensemble_learning) method to construct a multitude of [decision trees](http://en.wikipedia.org/wiki/Decision_tree_learning) through random subspace selection [55]. Prediction is made by aggregating (majority vote for classiﬁcation or averaging for regression) the predictions of the ensemble. Random forest consists of many decision trees, and each tree votes for a class based on its own classification result. Each decision tree is generated by the C4.5 algorithm [56].
