# Supplementary material for: Diagnostic assessment by dynamic contrast-enhanced and diffusion-weighted magnetic resonance in differentiation of breast lesions under different imaging protocols
Source: BMC Cancer. 2014 May 24;14:366. doi: 10.1186/1471-2407-14-366 (PMC4036635; doi:10.1186/1471-2407-14-366)
Supplement: Additional file 3 — Feature selection model of LHR. A simple explanation of the feature selection model of LHR. [file 1471-2407-14-366-S3.docx]

***Appendix 3. Feature weighting via Local Hyperplane based RELIEF (LHR)***

The RELIEF algorithm has been successfully applied in feature weighing due to

its simplicity and eﬀectiveness [46]. The main idea of Local Hyperplane based RELIEF model is to iteratively estimate the feature importance via local pattern approximation. There are two main steps involved in LHR, local hyperplane approximation and weighting estimations.

In the phase of local approximation, an observed sample$x$is firstly represented by a local hyperplane of class $c$ by:

$$LH_{c}(x)\text{ }=\text{ }\{s|s\text{ }=H\alpha\}\text{ }$$

where$H$is a $I\times n$ matrix composed by$n$NNs of the sample$x$:$H=\{h_{1},h_{2},\cdots,h_{n}\}$, with $h_{i}$being the i-th nearest neighbor of class $c$. The vector of $\alpha$ is the weights of the neighbors and is solved by minimizing the distance between the sample

$x$to its local hyperplane of $LH_{c}$:

$$\begin{matrix} J_{c}(\alpha)=\mathrm{argmin}\frac{1}{2}\sum_{i=1}^{I} \omega(i)(x_{i}-s_{i})^{2}=\frac{1}{2}(x-H\alpha)^{T}W(x-H\alpha) \\ \text{Subject to:} \\ \text{ }\sum_{i=1}^{k} \alpha_{i}=1,\alpha\geq0. \end{matrix}$$

where $s=H\alpha\in LH_{c}(x)$. $W$is a diagonal matrix with diagonal elements being the weight of the *i*-th feature.

In the phase of feature estimation, the feature importance is estimated via an maximization of margins:

$$\max_{W}E[W]=\frac{1}{N}\max_{W}\sum_{n=1}^{N} (\sum_{i=1}^{I} |x_{n}^{(i)}-LH_{NM}^{(i)}(x_{n})|-\sum_{i=1}^{I} |x_{n}^{(i)}-LH_{NH}^{(i)}(x_{n})|)$$

where$LH_{NM}^{(i)}$and $LH_{NH}^{(i)}$are the nearest neighbors for the sample $x^{(i)}$sharing the sample labels and different labels, respectively.

The aforementioned two phases are interactively updated until convergence. It has been shown in [45] that the LHR achieved nice performances when combined with various classification models.
